# Advancing Gender Equality in Healthcare Leadership: Protocol to Co-Design and Evaluate a Leadership and Mentoring Intervention in Tanzania

**DOI:** 10.5334/aogh.4374

**Published:** 2024-03-28

**Authors:** Doreen Mucheru, Henry Mollel, Brynne Gilmore, Anosisye Kesale, Eilish McAuliffe

**Affiliations:** 1 UCD School of Nursing, Midwifery and Health Systems, University College Dublin, Ireland; 2 Mbeya Campus College, Mzumbe University, Tanzania

**Keywords:** Leadership, healthcare, gender equality, gender equity, interventions, programmes, women

## Abstract

**Background:**

Women constitute almost two thirds of the health and social workforce. Yet, the proportion of women in decision-making positions remains significantly low leading to gender inequities in access to and appropriateness of healthcare. Several barriers which limit women’s advancement to leadership positions have been documented and they generally constitute of gender stereotypes, discrimination and inhibiting systems; these hinderances are compounded by intersection with other social identities. Amelioration of the barriers has the potential to enhance women’s participation in leadership and strengthen the existing health systems.

**Objective:**

This protocol describes a proposed study aimed at addressing the organisational and individual barriers to the advancement of women to leadership positions in the Tanzanian health sector, and to evaluate the influence on leadership competencies and career advancement actions of the female health workforce.

**Method:**

The study utilises a gender transformative approach, co-design and implementation science in the development and integration of a leadership and mentorship intervention for women in the Tanzanian health context. The key steps in this research include quantifying the gender ratio in healthcare leadership; identifying the individual and organisational barriers to women’s leadership; reviewing existing leadership, mentorship and career advancement interventions for women; recruiting programme participants for a leadership and mentorship programme; running a co-design workshop with programme participants and stakeholders; implementing a leadership and mentorship programme; and conducting a collaborative evaluation and lessons learnt.

**Conclusions:**

This research underscores the notion that progression towards gender equality in healthcare leadership is attained by fashioning a system that supports the advancement of women. We also argue that one of the pivotal indicators of progress towards the gender equality sustainable development goal is the number of women in senior and middle management positions, which we hope to further through this research.

## Introduction

A report published by Women in Global Health earlier this year – *The State of Women and Leadership in Health* – sets out the gender leadership gap in the global health workforce [1]. The report underlines that 70% of health workers are women but they only occupy 25% of leadership roles – this is the “xx paradox” which highlights striking inequity [1]. This leadership gap is ironic given that women constitute a majority of health and social care workforce [2]. The absence of women in leadership is owing to a labyrinth of visible and invisible barriers [3]. Barriers are consequential to power imbalances, gender stereotyping, discrimination and the dominance of structures which favour one gender [2]. These factors are not limited to a ‘glass ceiling’ which was the former narrative but a faulty system [4]. A commentary in the Lancet by Horton [5] opposes the notion that a new generation of women will inevitably rise to leadership given time and patience. He warns that the contrary is likely, because of the ‘broken pipeline’ between female health workers and the global health leadership. The faulty system of politics and patronage favours men, excludes women and is unlikely to yield gender equality in leadership [5].

Within the global health ecosystem, women experience disempowering environments, typified by gender stereotypes and prejudices which inhibit leadership advancement [2]. Women are erroneously discounted as leaders owing to the association of leadership with masculinity, and are viewed negatively when they demonstrate leadership traits [6]. The schism in leadership is compounded by occupational segregation where men are more likely to take up more prestigious and profitable careers such as medicine, which perpetuates the leadership disparity [2]. Therefore, staff within the nursing profession – often women – are less included in healthcare leadership [2]. Further, bullying and sexual harassment is often directed at women which impedes career advancement due to increased stress, decreased morale and productivity [7]. This is in conjunction with the lack of recognition that contributes to career stagnation – women are awarded less, their voices are less represented and respected and they are disproportionately invested in and supported [8,9]. This feeds into the cycle of insufficient women in healthcare leadership which inadvertently widens the pay gap [10].

Gender identity represents one dimension of obstacles that women contend with [11]. Marginalisation within healthcare leadership is the result of intersections between social identities including race, ethnicity, disability, class, religion, age and sexual orientation which are embedded in social systems [11].

Rectification of healthcare leadership gaps has the potential to strengthen existing health systems [2]. It is projected that greater inclusion of women will empower and motivate the workforce, improve the quality of care, and reduce attrition [2]. Women are credited for their transformational leadership skills linked to motivation, team advancement, individual emphasis, and inspiring hope for the future [2]. From an economic vantage point, attainment of gender parity in healthcare leadership will increase annual global gross domestic product by up to $28 trillion, which is about 26% of current GDP [12,13]. For perspective, this equates to the merged economies of the United States and China [12,13]. More conservatively, $12 trillion could be added to the global GDP if all countries matched their best-in-region country for gender parity – which is already twice the growth of the current ‘business as usual scenario’ [12,13]. This estimate does not account for the monetization of the unpaid care work, of which, women provide about three quarters globally [12,13].

Global policy acknowledges the importance of including women in healthcare leadership [14]. Gender equality and empowerment of all women and girls is one of the United Nations (UN) Sustainable Development Goals (SDGs); these goals aim to enhance health, justice and prosperity for all [14]. The UN also recognises that gender equality is a human right [14]. The gender equality sustainable development goal targets the full and effective participation of women, along with the availability of opportunities for leadership at all decision-making levels in all spheres of political, economic and public life [15].

Notwithstanding, women’s status in global health leadership has remained unchanged over the last five years, enumerating just 25% of senior leadership [1]. That said, the proportion of Fortune 500 healthcare companies led by women has increased from 5% to 12% but the number of female ministers of health has decreased from 31% to 25%, with only a fifth of government ministers being women [1,16]. Between 2018 and 2022, the proportion of World Health Assembly (WHA) delegations led by women fell from 27% to 23% [1]. Women in Global Health analysis reveals that 83% of delegations to the World Health Assembly (WHA) over the previous seven decades were primarily composed of men, and no WHA comprised of more than 30% of women chief delegates [1]. Research from WGH predicts that some countries may take over 100 years to attain gender parity in their WHA delegations based on current trends [17]. Attainment of gender equity in global health leadership, requires stronger political backing at the country level to generate the political capital required for the sponsorship of government and multilateral leadership roles [1].

In Tanzania, the National Policy on Women Development and Gender (NPWDG), approved the National Sub-Program for Women’s and Gender Advancement in 2000 as a national strategy to implement the Beijing Platform of Action [18]. This Sub-Program addresses four of the 12 critical areas of concern identified in the Beijing Platform for Action: Enhancement of women’s legal capacity; economic empowerment of women and poverty eradication; enhancement of women’s political empowerment and decision-making; and women’s access to education, training and employment [19].

The broad objective of the NPWDG is to guide sectors and institutions to ensure that gender competent plans and strategies are developed [18]. The NPWDG places emphasis on gender equality and the development of indicators for measuring gender equality in national initiatives [18]. The National Strategy for Gender and Development (NSGD) highlights major issues of concern to gender equality, and provides guidance on appropriate interventions and the roles of various actors and stakeholders in achieving these [18]. The NSGD calls for gender mainstreaming in all policies, programmes, plans, strategies, budgets and activities to bridge the existing gender gaps [18]. Moreover, availability of gender disaggregated data and provision of guidelines that will enforce this is required [18]. Despite Tanzania’s ratification of International Conventions and Regional Declarations that reflect a commitment to protect women’s human rights and address gender inequalities in leadership, the proportion of females in key decision-making positions at the different levels of both the public and private sectors, including the health sector is decreasing [20]. Hence the rationale for this study that aims to address the organisational and individual barriers to the advancement of women to leadership positions in the Tanzanian health sector.

### Framework for change

Leadership advancement among women is a product of imparting the necessary skills, leveraging their strengths and providing resources to circumnavigate barriers, embedded in a setting where organisational and systemic change facilitates and accommodates women’s leadership [21]. Effective leadership interventions that progress women’s leadership are generally characterised by an organisational component that is channelled through changes in organisational processes, creation of awareness, mentoring and networking, leadership training, support tools and resources [22]. Closing the leadership gap in health can only be done by addressing the systemic barriers women face [10]. A policy action paper by the World Health Organization, the Global Health Workforce Network and Women in Global Health, proposed a framework for change in supporting women’s leadership [10]. The framework emphasizes the centrality of addressing workplace systems and culture, and the use of deliberate measures to enable women to achieve [10]. Addressing workplace systems and culture to promote the leadership of women can be done by visible and accountable senior leadership, targets and quotas for gender parity in leadership, sensitisation of men to engage with and lead gender transformation, gender-transformative and retention strategies and adoption of an equality and family friendly policy [10]. Conversely, strategies which empower women to lead comprise development of networks for leadership advancement, increasing public visibility of women in decision-making, tracking and publishing key metrics on representation, developing peer support mechanisms and mentoring women [10].

### Aims and objectives

Against this backdrop, this study aims to implement a package of interventions to address the organisational and individual barriers to the advancement of women to leadership positions in the Tanzanian health sector, and to evaluate the influence on leadership competencies and career advancement actions of the female health workforce. More concisely, the project seeks to address gendered leadership by advancing systems and structures through collective leadership, networks, and mentorship. Specific objectives of this project are to:

- Determine the current gendered leadership characteristics and barriers in healthcare across three selected regions in Tanzania.
- Co-design and implement a mentorship, networking and leadership support programme for women healthcare workers across the three Tanzanian sites.
- Document and share programme resources, learning and best practices for supporting women’s leadership in healthcare.

## Methods

The project will apply a gender transformative approach, co-design and implementation science in the development and integration of a leadership and mentorship intervention for women in the Tanzanian health context.

### Gender transformative research

Gender transformative research (GTR) aims to foster gender equality and empower women and girls as a strategy to transform gender power dynamics and aligns relevant structures within communities and societies [23]. The proposed work will not only implement a programme for female healthcare workers but also garner support from government officials and sectors, with the purpose of confronting the intrapersonal and structural barriers which disempower women’s leadership.

Gender transformative research explores research questions pertinent to social norms, attitudes, behaviours and social systems that support gender inequality [23]. This approach actively engages groups in the process of critically examining, challenging and scrutinising gender norms and power relations [23]. Gender stereotypes and biased practices in the workplace will be assessed in realisation of this GTR principle.

Emerging insights on efficient utility of a gender transformative approach that will be incorporated in the research include clear and initial understanding of the context, getting men and women to jointly engage in critical reflection and identify options for change and building strong relationships between critical actors such as researchers, government officials, men, women and youth in local communities [24]. Finally, all data collected will be disaggregated by sex [25].

### Co-design

Co-design is the involvement of multiple stakeholders in the design and development of products, services or systems, with the goal of creating relevant, effective and acceptable solutions for the end-user [26]. This process goes beyond user consultation but partners with stakeholders as co-creators, where community and user experiences are at the core of the design process [26]. Co-design is an empowerment mechanism for individuals and communities to develop ideas, knowledge and skills in the resolution of issues that influence their lives [27].

Central to co-design is that project outcomes are agreed upon following in-depth analysis of the context, and research aims are aligned to policy [28]. Co-design captures experiences of patient and healthcare staff, ensuring that researchers, leaders and policy makers understand the reality and challenges faced by service users and deliverers [26]. There is accumulating evidence that co-design is likely to result in more sustainable change [28]. Co-design is particularly suited to studying gender and for gender transformative research, which emphasises participant critical engagement and contribution.

### Implementation science

Implementation science is the scientific study of methods that promote uptake of research, which is distinct from seeking to establish efficacy of a clinical innovation [29]. The methodology aims to pinpoint barriers and facilitators across various levels in the implementation context, and thereafter apply strategies to address the barriers and enhance factors which facilitate uptake of the innovation [29]. Notably, implementation science actively engages with the context pertinent to the innovation to unveil the optimal implementation strategy [29]. In implementation research, operational partners such as healthcare system leaders, administrators and healthcare providers are full partners in the research [29]. The project at hand will engage with these actors at various stages including, research design, intervention co-creation, participant recruitment, implementation and sustainability conception.

### Context of the research

**Figure 1:**
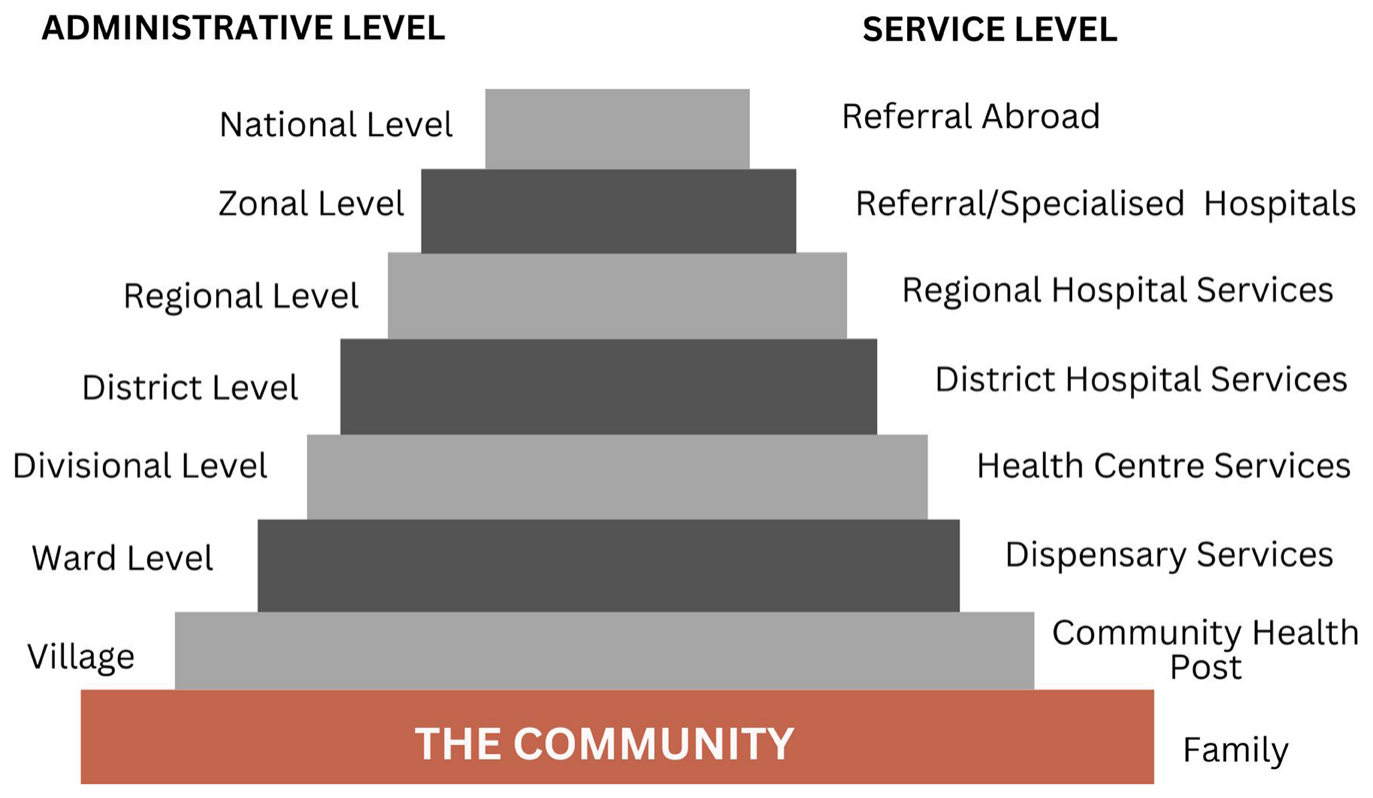
Tanzania’s Healthcare System and Corresponding Administrative System [31].

In Tanzania the health system structure parallels the political-administrative hierarchy [30] (see Figure 1). National Hospitals are the highest specialised referral point in the country; secondary to this are zonal hospitals which serve the five zones [30]. Both national and zonal hospitals are generally teaching hospitals and provide complex healthcare which requires advanced technology and skilled personnel [30]. The regional referral hospitals service the 26 Tanzanian regions [30]. Regional hospitals provide a wide range of services including specialised care, and public health preventative programs [30]. Regions are further subdivided into districts which are served by district hospitals [30]. Regional hospitals differ from the district hospitals because they are larger and offer more specialised care [30].

Health centres resource the division level and are primarily for preventative healthcare and treatment of simple medical conditions, but in some cases, provide reproductive health services and minor surgery [30]. Wards succeed the division administration rank and dispensaries are available at this level. Dispensaries offer basic outpatient care [30]. At the base of this political structure are villages which are served by a community health practitioner who provides health education and basic treatment to people in their homes [30].

### Tanzanian study sites

**Figure 2:**
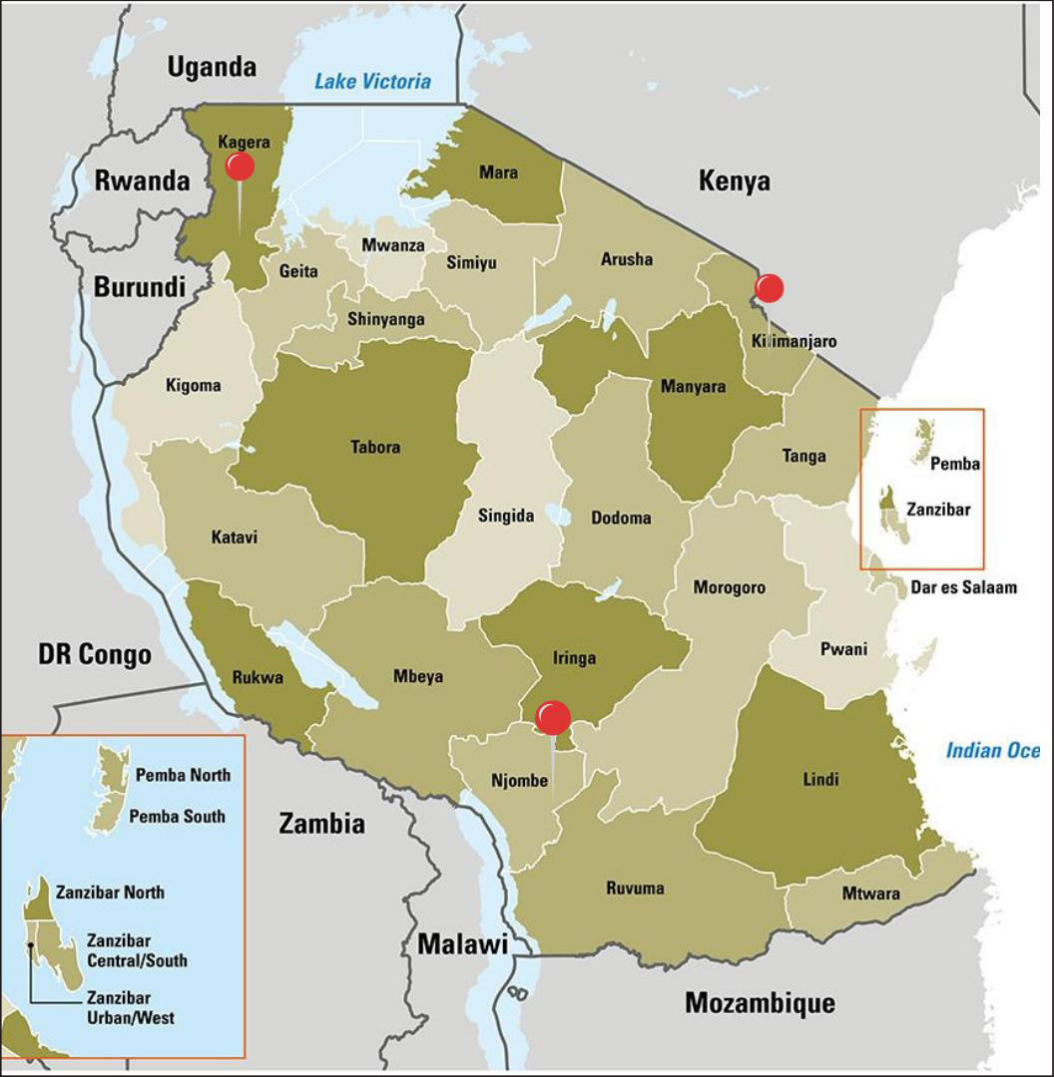
Research Regions [32].

Selection of project sites was based on the proportion of female and males in leadership within Tanzanian regions. High levels of disparity in the numbers of males and females in leadership suggests the presence of barriers that inhibit advancement of women to healthcare leadership, thus regions demonstrating this criterion were selected. The top three regions with the highest disparity in ratio of male to female leadership were Njombe, Kilimanjaro and Kagera (see Figure 2). A minimum of two councils were selected from each region, ensuring representation from urban, semi-urban and rural councils. Councils selected in each of the regions are:

- Njombe region – Njombe Town Council and Makete District Council
- Kagera region – Bukoba Municipal Council and Ngara District Council
- Kilimanjaro region – Moshi Municipal Council, and Same District Council

Mwanza region was selected as a comparison site as it is an urbanised region with moderate variation.

From each selected council, three health centres were purposefully identified, each from an urban, semi-urban, rural or peripheral setting. Therefore, 27 health centres will be included in the research. Four to five women will be selected from each health centre based on their willingness and eligibility to participate.

### Approach

The project will be implemented in four phases to allow a logical and comprehensive analysis of organisational and individual barriers to the advancement of women in leadership positions as follows:

#### Phase one

The key steps in this research phase are to:

- Determine the ratio of male and female staff in healthcare leadership positions across all Tanzanian regions.
- Identify the organisational and individual barriers to women’s leadership across the selected four research regions.
- Characterise prevailing workplace gender stereotypes and gender equality in healthcare across Tanzanian regions.
- Recruit healthcare leaders interested in participating in the second research phase as mentors from all Tanzanian regions.

Phase one is a descriptive, cross-sectional analysis of gender equality.

##### Leadership gender ratio

Data on leadership from regions, councils and districts will be sought by requesting administrative record access. Data pertaining to the role of leadership personnel in regions, councils and districts and their gender will be retrieved. An excel spread sheet will be distributed to government officials in the Tanzanian context for completion and returned to the research team for analysis.

Quantitative data will be analysed using Excel software by running descriptives.

##### Barriers to women’s leadership

Barriers to women’s leadership will be identified through interviews. The interviews will target healthcare workers, health managers and policy makers. A separate interview guide will be used for each of these groups. Purposeful selection of participants will be applied based on their eligibility and willingness to participate. Key themes of interest are leadership aspirations of healthcare workers, their experience with mentorship, differences between men and women as healthcare workers and opportunities available to them, workplace challenges experienced by women, presence of strategies to support women in overcoming obstacles at work, and processes and policies for promotion. The interview guide was developed by a gender expert with feedback from Tanzanian stakeholders regarding contextual relevance, and thereafter translated to Kiswahili – the local language – by team members.

Thematic analysis will be applied for the qualitative data using NVivo.

##### Workplace gender stereotypes

The prevailing workplace gender stereotypes and gender equality will be assessed using a brief survey targeted at healthcare leaders and managers across all regions in Tanzania. The tool will be based on validated tools on gender stereotypes and equality, in tandem with stakeholder input. The tool will be distributed as a google form and piloted on a small sample of leaders and managers.

Recruitment of healthcare leaders to participate in subsequent phases of the project that involve mentoring will also be achieved using the aforementioned google form. The tool will be distributed via email and WhatsApp mediums by local partners who have access to the staff directory. The survey is intended to recruit as many healthcare leaders and managers as are willing to participate.

Quantitative data will be analysed using SPSS software where relevant descriptives and tests of association will be run.

##### Rapid realist review on leadership and career advancement interventions

To further support this initial phase of research, a rapid realist review is being conducted on leadership and career advancement interventions for women in healthcare [33]. This expedited realist review will help identify why certain programmes work in certain ways, for specific people and in particular circumstances through the illustration of a theory [33]. The theory articulates the link between the interventions, contexts for interventions, participant responses and intervention outcomes which may not be clearly evident and articulated by a conventional review of the literature [33]. An expert panel who as the name suggests, comprise of authorities in the field of leadership, gender and healthcare as well as practitioners from the Tanzanian context, will be engaged to provide feedback on relevance of the theories generated from the review process [33]. The final theories generated will contribute to the development of a leadership and career advancement intervention for women in the Tanzanian healthcare setting [33].

#### Phase two

The key steps in this research phase are to:

- Recruit 120 female healthcare workers as participants and 30 influential, networked male leaders across the three sites and 30 female leaders.
- Conduct a baseline survey with the 120 female healthcare workers and healthcare leaders of whom 30 are male and 30 are female to determine reference characteristics.
- Run a codesign workshop with participants and stakeholders to design a leadership and mentorship programme for women in the Tanzanian healthcare setting.

##### Recruitment

Recruitment of the 120 female healthcare workers will be facilitated by team members who are based in Tanzania. Team members will visit the research sites where they will hold informal workshops detailing the aims and objectives of this research. All female healthcare workers who were willing to participate in the baseline survey as well as subsequent phases of the research will be recruited into the study. Research team members will aim for recruitment of about 30% of participants from each of the three research regions to ensure even distribution of participants.

The 30 male and 30 female healthcare leaders will be recruited through the same process where research team members will detail the aims and objectives of the research and invite them to take part in the baseline along with subsequent phases of research.

##### Baseline surveys

The baseline survey will target female healthcare workers and seek out information on demographics (including marital status, having children, age of children levels of social support), workplace role, gender bias in the workplace, leadership evaluation, leadership self-efficacy, relationship with workplace leadership, networking participation and burnout. The survey will be developed using validated scales and Tanzania stakeholders will also provide feedback on considerations that would enhance the contextual relevance. Additionally, leadership skills and competencies will be identified from local guidelines for healthcare workers and feedback from Tanzanian stakeholders. The survey will be piloted and translated into Kiswahili.

The baseline survey for the male and female healthcare leaders will acquire information on demographics, gender bias in the workplace, gender equality in the workplace and evaluate initial leadership attributes among the female healthcare workers. Validated scales will be used to develop the survey, with stakeholder feedback on contextual relevance of the tool. The survey will be piloted and translated into Kiswahili.

Both baseline surveys for the female healthcare workers and healthcare leaders will be delivered by team members concurrently with recruitment. A mobile data collecting (MDC) quantitative approach will be implemented by the research assistants. Data will be collected via mobile phones where research assistants facilitate completion and submission of the survey. Data will then be transmitted to a central server. Anticipated completion-time is 15 to 25 minutes.

Quantitative data will be analysed using SPSS software while qualitative data will be analysed using Nvivo software. Objectives of quantitative data analysis will include obtaining descriptive statistics, identifying patterns of gender bias and stereotypes to inform intervention design, and comparing outcomes pertaining to leadership, networking and wellbeing before and after the intervention. Qualitative data analysis will facilitate identification of nuances related to workplace realities, gender inequalities and leadership barriers for women which will also inform the intervention.

##### Co-design workshop

This codesign aspect of the research will involve healthcare workers, healthcare leaders and managers, policy makers, academics and government officials from the Tanzanian context to inform the design of the leadership and mentorship programme or female healthcare workers [27].

### Selection criteria for co-design participants

- Employed as a healthcare worker, such as doctor, nurse or medical officer in the three Tanzanian research regions or;
- Employed as a healthcare leader or manager whose role entails overseeing or supervising healthcare workers in the three Tanzanian research regions or;
- Policy makers such as members of parliament or members of the local government such as mayors and councillors who oversee city, municipal and town councils or;
- Government officials such as permanent secretaries or deputy permanent secretaries who oversee government ministry of health day-to-day activities, and health secretaries who oversee health administrative duties across regions, councils and districts or;
- Academics who are core team members or affiliated with the project.

The team will endeavour to have two participants from each of the identified categories, with a gender ratio of about 50% men and 50% women. The project is endorsed by the President’s Office Regional Administration and Local Government Tanzania (PO-RALG) which is the body that interprets and implements policy, along with managing district and regional health services. President’s Office Regional Administration and Local Government Tanzania will support the codesign process by inviting government healthcare workers, managers, and policy makers to share their input because the project aims parallel government policy objectives.

Principles that will underpin the co-design process include mutual respect between the research team and co-design partners, inclusivity, flexibility, accountability of the research team, fairness in participation opportunities, and an active participation process [27].

The Generative Co-Design Framework for Healthcare innovation will guide the co-design process [34]. This framework breaks-down codesign into three phases [34]. The pre-design phase commences with contextual inquiry where there the setting of interest is explored [34]. This will be achieved through baseline interviews and surveys mentioned in phase one and two of the research [34]. The next step within the predesign phase is development of informational material for participants to refer to during co-design, selection of participants and facilitators and technology tests [34]. Development of materials for this step is contingent upon analysis of data from phase one and two of the research [34].

The core co-design phase involves sharing ideas with co-design partners such as the design of the prospective innovation, identifying explicit as well as the latent participant needs and thereafter communicating a vision of the work alongside anticipated changes [34]. Specific foci for this co-design phase include specific mentor and mentee roles, associated responsibilities and procedures, identifying training requirements, frequency and timing of programme sessions, project outcome indicators and co-evaluation procedures. The phase that follows this is the post-design phase where data from the active co-design will be analysed, and thereafter theme checking with co-design participants to ensure that the most significant and pertinent ideas are captured [34]. In the final step, the research team and co-design participants identify priorities from the co-design phase and plan for the intervention implementation stages [34].

#### Phase three

The key action for this research phase is to implement a leadership and mentorship intervention for female healthcare workers in the Tanzanian health sector.

##### Leadership and mentorship intervention

The final leadership and mentorship intervention for female healthcare workers in the Tanzanian setting will be based on findings from the rapid realist review, baseline surveys and interviews, and co-design process.

The projected intervention will take a collaborative leadership development approach, including tripartite mentorship. The three elements of tripartite mentorship will encompass mentorship from a male influential leader, mentorship from a senior female leader and peer mentorship support.

**Figure 3:**
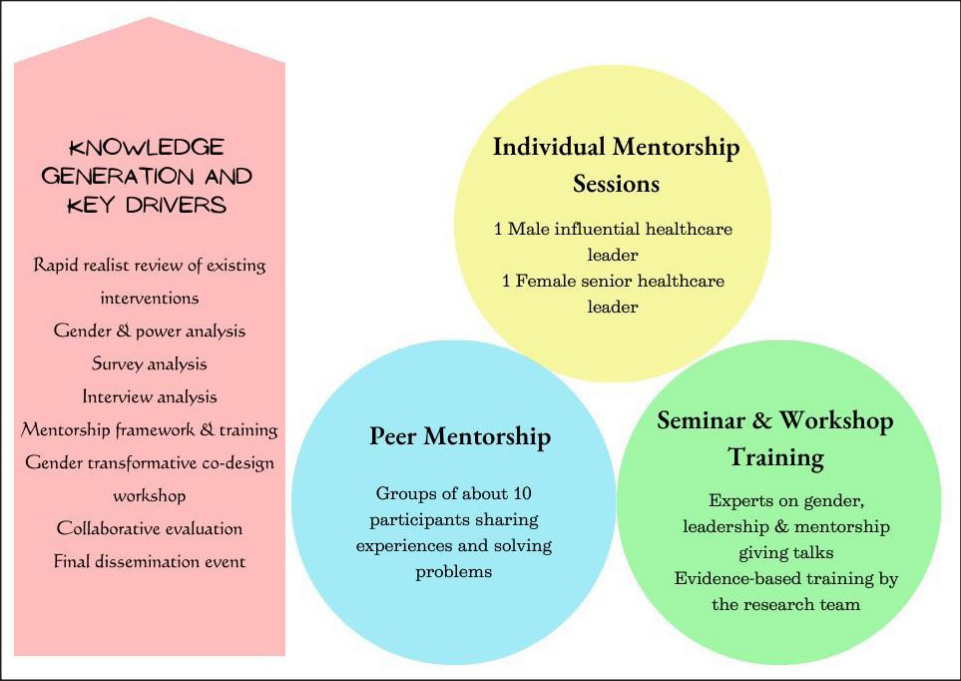
Leadership and Mentorship Blueprint.

Leadership training will comprise seminars and workshops delivered by experts (contingent on participants’ needs) to support improved understanding and knowledge of gender and leadership competencies. Ultimately, the seminars and workshops will aim to provide participants with the necessary knowledge and tools to develop their personal leadership style and utilise their strengths to become transformative leaders (see Figure 3).

#### Phase four

The key actions for this research phase are to:

- Conduct endline surveys with all female healthcare workers to assess changes in leadership competencies.
- Conduct endline surveys with female healthcare worker’s line managers to explore any changes in perceptions of participant’s competencies and leadership attributes and understand perceived impact of the intervention from the management perspective.
- Collaborative evaluation with research team, select participants and mentors to assess alignment with the RE-AIM Framework
- Document and share programme resources, learning and best practices for supporting women’s leadership in Tanzanian healthcare.
- Collaborative shared lessons learned and recommendations for future implementation and scale-up will be conducted based on evaluation of findings.

##### Endline surveys

The endline survey with female healthcare workers will mirror the baseline to evaluate changes in leadership skills, leadership self-efficacy, networking participation and burnout. The survey will target all female healthcare workers who complete the leadership and mentorship programme. Similarly, the endline survey for healthcare leaders will evaluate perception of changes in leadership for the participating female healthcare workers using the same scales applied in the baseline.

##### Collaborative evaluation and shared lessons

The significance of the intervention will be evaluated using the RE-AIM framework which assesses the significance of public health, community-based, system-based and socio-ecological interventions [35]. This framework conceptualises the impact of an intervention based on the paradigms of reach, efficacy, adoption, implementation and maintenance [35].

Reach is measured by comparing actual programme participants versus all potentially eligible members of a defined population [35]. Efficacy is evaluated by both the positive and negative outcomes of a programme [35]. Adoption as per the framework refers to the proportion and representativeness of settings that take up the policy or programme in question [35]. Implementation is the extent which a programme is delivered as proposed while maintenance denotes long-term continuation with the behaviour change, owing to programme implementation [35]. These variables will be evaluated using surveys and interviews which target healthcare workers and leaders, and by comparing characteristics of participating healthcare workers, leaders and regions, with the those that were eligible but did not engage with the intervention.

## Discussion

This research project aims to implement a package of interventions to address the organisational and individual barriers to the advancement of women to healthcare leadership positions in Tanzania, evaluate the efficacy, and share lessons learnt from the programme. This research is unique because it underscores the concept of gender equality in a setting where execution is slow [36]. Reports from UN Women indicated that by the end of 2020, less than 50% of sustainable goal indicators from a gender perspective were available for the Tanzanian population [36]. One pivotal indicator of progress towards the gender equity sustainable development goal, is the number of women in senior and middle management positions [37]. This project seeks to publish data pertinent to this within the healthcare system in Tanzania [2].

Effective and sustainable inclusion of women in leadership must involve gender responsive leadership at all levels of health systems, and development of enabling environments for women’s leadership within organisations by those with authority to do so [38]. The project appropriates these strategies by targeting potential female leaders in the early and mid-stages of their careers. Moreover, the participation of policy makers, healthcare leaders and managers in setting the agenda and delivery of various programme components supports a system approach so that organisational change is instigated.

The research project incorporates a unique mentoring approach where each participant accesses a male and female mentor. Research highlights that having a female mentor mainly supports protégés through role modelling whereas males provide prospects for career advancement [39]. Since women are more likely to encounter interpersonal and organisational obstacles to progression, career advancement functions may improve their chances of promotion, higher income and workplace benefits [39].

The extensive evaluation of the intervention through application of the RE-AIM framework will highlight the programme’s reach, adoption, effectiveness, implementation and maintenance which is extremely relevant to the busy, understaffed public health and wider health system [35]. This holistic evaluation will provide insight on individual and organisational performance as concerning the reach, positive and negative outcomes, representativeness of participating settings and sustainability of the programme [35]. This information will be extremely seminal for successive scale-up of the effective programmatic components.
